# IL-6/STAT3 Signaling Promotes Cardiac Dysfunction by Upregulating FUNDC1-Dependent Mitochondria-Associated Endoplasmic Reticulum Membranes Formation in Sepsis Mice

**DOI:** 10.3389/fcvm.2021.790612

**Published:** 2022-01-18

**Authors:** Tao Jiang, Dewei Peng, Wei Shi, Junyi Guo, Shengqi Huo, Lintong Men, Cuntai Zhang, Sheng Li, Jiagao Lv, Li Lin

**Affiliations:** ^1^Division of Cardiology, Department of Internal Medicine, Tongji Hospital, Tongji Medical College, Huazhong University of Science and Technology, Wuhan, China; ^2^Department of Geriatrics, Tongji Hospital, Tongji Medical College, Huazhong University of Science and Technology, Wuhan, China

**Keywords:** sepsis-induced myocardial dysfunction, FUNDC1, MAMs, mitophagy, calcium signaling

## Abstract

**Aims:**

Cytokine storm is closely related to the initiation and progression of sepsis, and the level of IL-6 is positively correlated with mortality and organ dysfunction. Sepsis-induced myocardial dysfunction (SIMD) is one of the major complications. However, the role of the IL-6/STAT3 signaling in the SIMD remains unclear.

**Methods and Results:**

Septic mice were induced by intraperitoneal injection of LPS (10 mg/kg). Echocardiography, cytokines detection, and histologic examination showed that sepsis mice developed cardiac systolic and diastolic dysfunction, increase of inflammatory cytokines in serum, activated STAT3 and TLR4/NFκB pathway in heart, and raised myocardial apoptosis, which were attenuated by IL-6/STAT3 inhibitor, Bazedoxifene. *In vitro*, we found that LPS decreased cell viability in a concentration-dependent manner and activated STAT3. Western blot and immunofluorescence results indicated that STAT3 phosphorylation induced by LPS was inhibited by Bazedoxifene. Bazedoxifene also suppressed LPS-induced IL-6 transcription. sIL-6R caused LPS-induced p-STAT3 firstly decreased and then significantly increased. More importantly, we found STAT3-knockdown suppressed LPS-induced expression of FUNDC1, a protein located in mitochondria-associated endoplasmic reticulum membranes (MAMs). Overexpression of STAT3 led to an increase in FUNDC1 expression. Dual-luciferase reporter assay was used to confirm that STAT3 was a potential transcription factor for FUNDC1. Moreover, we showed that LPS increased MAMs formation and intracellular Ca^2+^ levels, enhanced the expression of Cav1.2 and RyR2, decreased mitochondrial membrane potential and intracellular ATP levels, and promoted mitochondrial fragmentation, the expression of mitophagy proteins and ROS production in H9c2 cells, which were reversed by knockdown of FUNDC1 and IL-6/STAT3 inhibitor including Bazedoxifene and Stattic.

**Conclusions:**

IL-6/STAT3 pathway plays a key role in LPS-induced myocardial dysfunction, through regulating the FUNDC1-associated MAMs formation and interfering the function of ER and mitochondria. IL-6/STAT3/FUNDC1 signaling could be a new therapeutic target for SIMD.

## Introduction

Sepsis is defined as the dysregulated host response to infection contributing to life-threatening organ dysfunction (1). There are 20–30 million patients worldwide diagnosed with sepsis and septic shock and over eight million lives lost (2). Sepsis-induced myocardial dysfunction (SIMD) is one of the major complications in clinical sepsis, which contributes to systolic and diastolic dysfunction of heart (3, 4). The mortality in sepsis patients with cardiac dysfunction is significantly higher than those without cardiac dysfunction (70–90% vs. 20%) (5). At present, available treatments are limited for SIMD, including early wide-spectrum antibiotic treatment and fluid resuscitation (3).

The serum cytokines of sepsis are significantly increased, including IL-6, IL-1β, and TNF-α (6). Inflammatory reaction triggered by cytokines is the main cause of sepsis-induced multiple organ system failure (7). However, the specific mechanism underlying the effect of cytokines on SIMD remains incompletely understood. IL-6 is remarkably correlated to the severity of sepsis (8) and the clinical trial of its receptor antagonists is currently underway in children's severe sepsis (NCT04850443). IL-6 forms a complex with IL-6 receptor (IL-6R) and co-receptor glycoprotein 130 (gp130), and then activates JAKs and STAT3. Phosphorylated STAT3 subsequently dimerizes, translocates to the nucleus, and regulates gene transcription (9, 10). Recent study (11) shows that STAT3 is activated in SIMD. However, the specific function and regulatory mechanism of IL-6/STAT3 pathway in SIMD remains unclear.

FUN14 domain containing 1 (FUNDC1) is a mitochondrial outer-membrane (MOM) protein, which is reported to interact with the endoplasmic reticulum (ER) protein inositol 1,4,5-trisphosphate receptor (IP3R) and plays an important role in the formation of mitochondria-associated endoplasmic reticulum membranes (MAMs) (12). FUNDC1-dependent MAMs are involved in ER/mitochondria calcium exchange, and affected mitochondrial function and reactive oxygen species (ROS) production. The pathological mechanisms of SIMD include disturbance of inflammation mediator, mitochondrial dysfunction, apoptosis, and calcium dysregulation (13). Hence, FUNDC1-dependent MAMs may play a key role in the occurrence and development of SIMD. So far, no relevant literature has been reported. Previous studies (10, 14) show that IL-6/STAT3 inhibitor improves transaortic constriction mouse cardiac dysfunction and ameliorates IL-6 induced mitochondrial dysfunction and ROS production in H9c2 cells. Therefore, we speculate that STAT3 may affect FUNDC1-dependent MAMs formation and leads to mitochondrial dysfunction and ROS production in SIMD.

Bazedoxifene has been approved by the Food and Drug Administration for the treatment of female patients with postmenopausal osteoporosis. We have previously reported that Bazedoxifene could inhibit phosphorylation of STAT3 and prevents chronic cardiovascular diseases (14, 15). However, the potential effect of Bazedoxifene in acute cardiovascular diseases remains unclear. Here, we challenged mice with LPS to construct sepsis model and evaluated the effect of Bazedoxifene. We also utilized H9c2 cells and AC16 cells to explore the specific regulatory mechanisms. Our results showed that Bazedoxifene attenuated LPS-induced cardiac dysfunction. Moreover, we found that IL-6/STAT3 signaling promoted myocardial injury by upregulating FUNDC1-dependent MAMs formation.

## Materials and Methods

### Animals and Reagent

All animal experiments were approved by the Animal Care and Use Committee of Tongji Hospital, Tongji Medical College, Huazhong University of Science and Technology. Eight-week-old male wild-type C57BL/6J mice (about 22 g) were purchased from the Jackson Laboratory. Mice were randomly divided into three groups: Control, LPS, and LPS+ Bazedoxifene (hereafter referred to as BAZ) groups (*n* = 10). Bazedoxifene acetate (HY-A0036, MedChemExpress, U.S.) was dissolved in a vehicle [10% dimethyl sulfoxide (DMSO), 40% polyethylene glycol 300 (PEG300), 5% Tween-80, and 45% PBS]. For BAZ group, the mice were pretreated with Bazedoxifene (5 mg/kg) for 3 days by intragastric administration. Control and LPS groups were administered with the vehicle in parallel. On the fourth day, mice of LPS and BAZ groups were treated with LPS (10 mg/kg; L2630; Sigma-Aldrich, U.S.) by intraperitoneal injection to induce the sepsis. Control mice were injected intraperitoneally with PBS. After LPS or PBS treatment, the body weights and temperature of mice were monitored every 3 h for up to 12 h.

### Echocardiography

Echocardiography was performed using a VINNO6 high-resolution imaging system (VINNO Corporation, China). Echocardiography was performed at baseline and after 12 h of LPS treatment. Briefly, mice were anesthetized with isoflurane (3% isoflurane for induction, and 1–2% isoflurane for maintenance). Then mice were positioned supine, and the chests of mice were shaved. We determined the left ventricular structure and function using M-mode, Doppler flow, and tissue Doppler imaging. M-mode echocardiography were captured from the parasternal short axis view, then Doppler flow and tissue Doppler imaging were obtained from the apical 4-chamber view. We used eight parameters to assess left ventricular systolic and diastolic function: left ventricular ejection fraction (LVEF), left ventricular fractional shortening (LVFS), left ventricular internal dimensions at end systole (LVIDs) and diastole (LVIDd), left ventricular volumes at end systole (LVESV) and diastole (LVEDV), E/A ratio, and E'/A' ratio. Each parameter was the mean of three cardiac cycles. After echocardiography, mice were sacrificed with carbon dioxide.

### Cytokine Measurements

The IL-6 mouse uncoated ELISA kit (88-7064; Invitrogen, U.S.), and mouse IL-1β uncoated ELISA kit (88-7013; Invitrogen, U.S.) were used to measure IL-6 and IL-1β levels in mouse serum according to the manufacturer's instructions.

### Immunohistochemistry

After animals were sacrificed, the hearts were fixed with 10% formalin, embedded in paraffin and sectioned. Immunohistochemistry was performed with antibody against p-STAT3 (Tyr705) (#9131, Cell Signaling Technology, CST) to detect the protein expression. Image capture was operated using the MShot microscope (Wuhan, China).

### Analysis of Apoptosis

TUNEL staining was performed to detect the apoptosis in cardiac tissue. TUNEL staining was performed using TUNEL apoptosis assay kit (KGA7062; KeyGEN BioTECH, Nanjing, China) following the manufacturer's instructions. Apoptosis index = (number of apoptotic cells/ number of total cells) × 100%.

### Cell Culture and Treatment

H9c2 cells and AC16 cells were acquired from the American Type Culture Collection (ATCC, Manassas, U.S.) and cultured in high glucose Dulbecco's modified Eagle's medium (DMEM, KeyGEN BioTECH, Nanjing, China) supplemented with 10% fetal bovine serum (FBS, Gibco, Waltham, U.S.) and 1% penicillin/streptomycin (Sangon, Shanghai, China) at 37°C with 5% CO (2).

For cell treatment, H9c2 cells were divided into four groups: control group; LPS group, H9c2 cells were treated with 1 μg/ml LPS for 12 h; BAZ group, H9c2 cells were pretreated with 5 μM Bazedoxifene for 1 h followed by LPS induction for another 12 h; Stattic group, H9c2 cells were treated with 5 μM Stattic for 2 h, then the culture medium was replaced with fresh medium containing LPS for another 12 h.

As to the experiments of soluble IL-6R (sIL-6R), H9c2 cells were incubated with LPS and sIL-6R (0, 2.5, 5, 10, 20 ng/ml) for 12 h.

### Cell Viability Assay

Cell viability was examined by CCK8 assay. Briefly, H9c2 cells (5,000) were counted using a Cellometer-Mini Automatic Cell Counter (Nexcelom Biosciences, Lawrence, USA) and seeded in 96-well plates (6-wells per group). Following treatment, H9c2 cells were incubated with 10% CCK8 (HY-K0301; MedChemExpress, U.S.) for 2 h at 37°C in the dark. The absorbance was determined at 450 nm.

### Quantitative PCR

Total RNA was prepared with a Hipure Total RNA Mini Kit (Magen, Guangzhou, China). ReverTra Ace qPCR RT kit (TOYOBO Co., Ltd., Osaka, Japan) was used to synthesize cDNA. Quantitative PCR was carried out using the SYBR green PCR master mix kit (TOYOBO Co., Ltd., Osaka, Japan) on the StepOnePlus realtime PCR system (ThermoFisher Scientific, CA, U.S.). Quantitation of IL-6 mRNA was normalized to GAPDH by the ΔΔCt method. The primers were rat IL-6: forward: 5′-ACTTCCAGCCAGTTGCCTTCTTG-3′, reverse: 5′-TGGTCTGTTGTGGGTGGTATCCTC-3′; rat GAPDH: forward: 5′-AGGTCGGTGTGAACGGATTTG-3′, reverse: 5′-TGTAGACCATGTAGTTGAGGTCA-3′.

### Immunofluorescence Staining

H9c2 cells were grown on coverslips for 12 h and exposed to treatments. After treatment, cells were washed with PBS and then fixed by immersion in 4% paraformaldehyde for 15 min at room temperature. After rinsing with PBS, cells were permeabilized and blocked with 0.5% Triton-100 and 5% goat serum in PBS for 1 h at room temperature. Coverslips were then incubated with primary antibodies against p-STAT3 (Tyr705) (#9145, CST) at 4°C, and followed by incubation with fluorescence secondary antibody for 1 h. Finally, the DAPI-containing anti-fluorescence quencher was used to seal the glass slides. Slides were then examined using the confocal microscope (Nikon, Tokyo, Japan).

### Western Blot

Cardiac tissue homogenates and the collected cultured cells were lysed in the RIPA lysis buffer containing 1 mM protease inhibitor and 1 mM phosphatase inhibitor for 1 h on ice. The lysates were centrifuged at 12,000 rpm for 20 min at 4°C, then the supernatant was collected. Protein concentration was measured with a BCA protein assay kit. Equal amounts of protein were loaded on 10% Bis-Tris gel and separated by SDS-PAGE. Proteins were transferred to PVDF membranes, and the membranes were incubated with primary antibody at 4°C overnight, including p-STAT3 (Tyr705) (#9145, CST), p-STAT3 (Ser727) (#9134, CST), STAT3 (#4904, CST), TLR4 (ab217274, Abcam), p-NFκB (Ser536) (#3033, CST), NFκB (#8242, CST), FUNDC1 (#49240, CST), BNIP3 (ab10433, Abcam), NIX (#12396S, CST), PINK1 (ab23707, Abcam), P-IP3R (Ser1756) (#3760, CST), IP3R (#3763, CST), Cav1.2 (ab84814, Abcam), RYR2 (19765-1-AP, Proteintech), GAPDH (#2118, CST). Horseradish peroxidase-conjugated secondary antibodies and ultra high sensitivity ECL kit (HY-K1005; MedChemExpress, U.S.) were used for protein detection which was operated on the ChemiDoc-It 510 Imager with VisionWorks software (Ultra-Violet Products Ltd., Cambridge, UK).

### Transfection

For siRNA transfection, H9c2 cells were seeded in 6-well plates for 12 h and transfected with control siRNA, FUNDC1 siRNA (siFUNDC1, Hippo Biotechnology Co., Ltd., Zhejiang, China), or STAT3 siRNA (siSTAT3, RiboBio Co., Ltd., Guangzhou, China) using Lipo2000 (11668019; Invitrogen, U.S.). H9c2 cells were treated with LPS for 12 h at 48 h post-transfection.

For plasmid transfection, AC16 cells were seeded in 6-well plates for 12 h and transfected with a control plasmid or STAT3 overexpressed plasmid using Lipo3000 (L3000015; Invitrogen, U.S.). AC16 cells were treated with LPS for 12 h at 48 h post-transfection.

### Luciferase Assays

JASPAR database (http://jaspar.genereg.net/) was used to predict the binding sites of STAT3 on FUNDC1 promoter. The 2.0-kb wild-type FUNDC1 promoter and the mutant FUNDC1 promoter were cloned into the pGL3-basic luciferase reporter vector (Promega Corporation, U.S.). Then the recombinant constructs were subjected to the co-transfection with pRL-TK plasmid expressing Renilla luciferase (Promega Corporation, U.S.) and STAT3 overexpressed plasmid into AC16 cells. Firefly and Renilla luciferase activities were measured at 48 h after transfection using a Dual Luciferase Reporter Gene Assay Kit (11402ES60; Yeasen Biotech Co., Ltd., Shanghai, China). Renilla luciferase was used for normalization.

### ER and Mitochondria Contact Analysis

H9c2 cells were incubated with Mito-Tracker Red CMXRos (C1035; Beyotime Biotechnology, China) and ER-Tracker Green (C1042S; Beyotime Biotechnology, China) for 30 min at 37°C, and washed with prewarmed Hanks' balanced salt solution (HBSS, C0219; Beyotime Biotechnology, China), then fixed with 4% paraformaldehyde for 2 min at 37°C. The contact of ER and mitochondria was observed with a confocal microscope (Nikon, Tokyo, Japan) using a 100x oil immersion lens. ER and mitochondria contact analysis was measured on Image J using the Pearson's correlation coefficient.

### Detection of Intracellular Ca^2+^

Intracellular Ca^2+^ was determined using the fluorescent probe Fluo-4 AM (S1060; Beyotime Biotechnology, China). After treatment, H9c2 cells were incubated with 2 μM Fluo-4 AM for 30 min at 37°C in the dark, and then washed with PBS. The fluorescence signal intensity was measured with a confocal microscope (Nikon, Tokyo, Japan).

### Measurement of Mitochondrial Membrane Potential (Δψm)

Δψm was determined by fluorescent dye JC-1 (PJC-110; Promotor Biological Co., Ltd., China). After treatment, H9c2 cells were incubated with JC-1 for 30 min at 37°C, then washed with PBS and covered with cell culture medium. The fluorescence signals of JC-1 aggregate (red, 525/590 nm) and JC-1 monomer (green, 485/530 nm) were detected with the MShot fluorescence microscope (Wuhan, China). Δψm was expressed as the ratio of JC-1 aggregate/ JC-1 monomer.

### ATP Assays

Intracellular ATP content was measured using ATP assay kit (S0026; Beyotime Biotechnology, China). Following treatment, H9c2 cells were lysed with equal amounts of cell lysis buffer, followed by centrifugation at 12,000 g for 5 min at 4°C. The supernatant was collected and reacted with the ATP detection working solution, and luminescence was determined using a GloMax luminometer (Promega Corporation, U.S.). The ATP content was calculated against the standard curves and standardized according to sample protein concentrations.

### Mitochondria Staining and Analysis of Mitochondrial Network Morphology

Mitochondria staining was performed following the manufacturer's instructions. Briefly, cells cultured in 6-well plates were incubated with 200 nM Mito-Tracker Red CMXRos (C1035; Beyotime Biotechnology, China) for 20 min at 37°C, and washed with prewarmed HBSS, then fixed with 4% paraformaldehyde for 15 min at 37°C. The mitochondrial network morphology of cells was observed with a confocal microscope (Nikon, Tokyo, Japan) using a 100x oil immersion lens. The mitochondrial network morphology was analyzed using the ImageJ macro tool MiNA. We used four parameters to evaluate the mitochondrial network complexity: percentage of network mitochondria, mean number of mitochondrial branches, mean branch length, and mitochondrial footprint.

### Assessment of ROS

ROS production was evaluated by fluorescent dye 2',7'-Dichlorodihydrofluorescein diacetate (H2DCFDA) (HY-D0940; MedChemExpress, U.S.). Following treatment, H9c2 cells were incubated with 10 μM H2DCFDA for 30 min at 37°C protected from light, and then washed with prewarmed HBSS, then fixed with 4% paraformaldehyde for 15 min at 37°C. The fluorescence intensity was measured with the confocal microscope (Nikon, Tokyo, Japan).

### Statistical Analysis

SPSS 26.0 and GraphPad 8.0 were used for statistical analysis and drawing. Data from experiments were presented as mean± S.E.M. Statistical analysis was performed using unpaired Student's *t*-test, one-way analysis of variance (ANOVA) or two-way ANOVA with Bonferroni's *post-hoc* test. Quantitative assessments were performed with Image J (Fiji) software. Statistical significance was defined as *p* < 0.05.

## Results

### Bazedoxifene Attenuated Cardiac Dysfunction in LPS-Induced Sepsis

**Figure 1:**
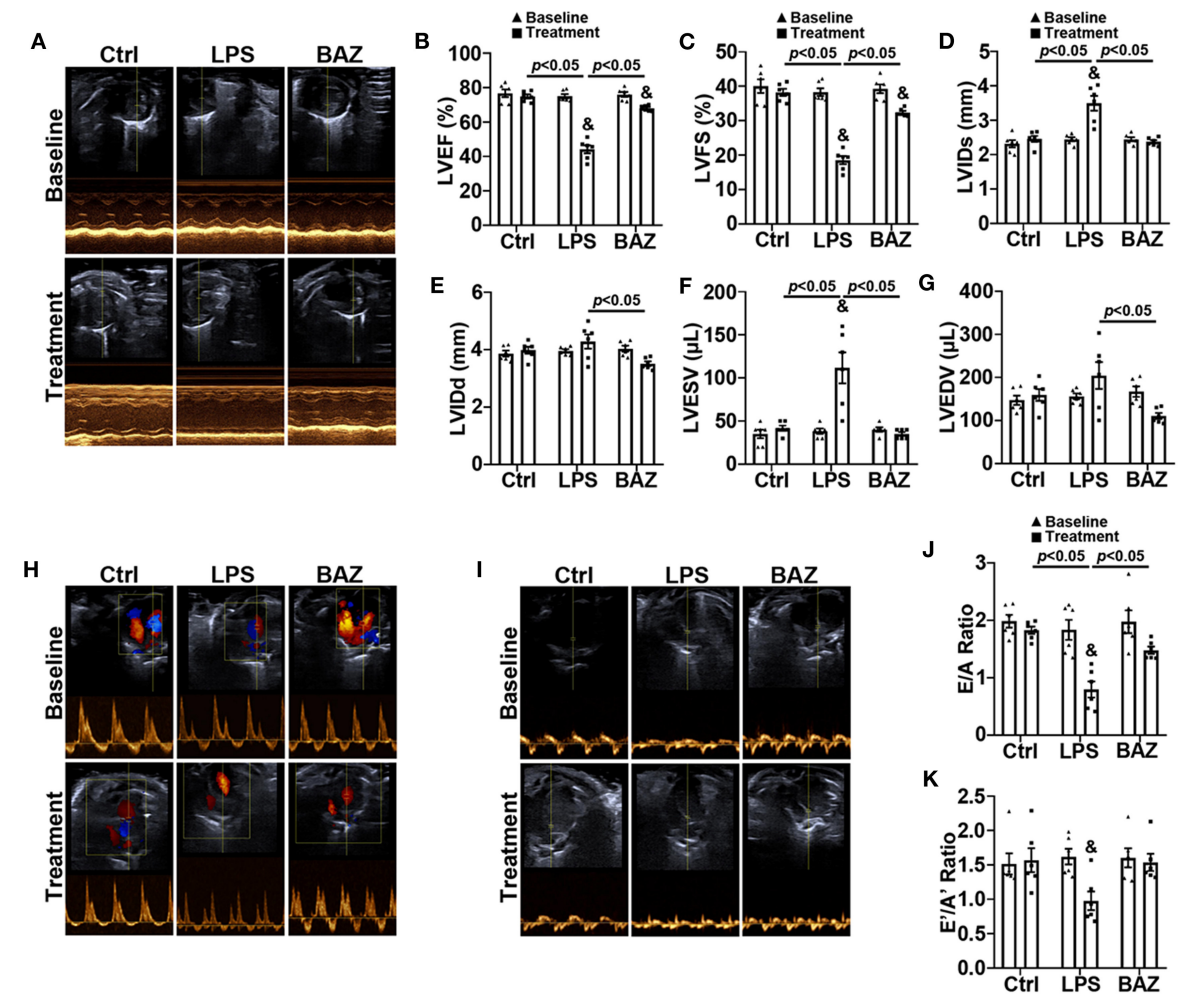
Bazedoxifene ameliorated systolic and diastolic dysfunction in LPS-challenged mice. **(A)** Representative left ventricular echocardiographic M-mode images before and 12 h after LPS treatment. **(B–G)** Mean data of left ventricular ejection fraction (LVEF) **(B)**, left ventricular fractional shortening (LVFS) **(C)**, left ventricular internal dimensions at end systole (LVIDs) **(D)** and diastole (LVIDd) **(E)**, left ventricular volumes at end systole (LVESV) **(F)** and diastole (LVEDV) **(G)**. **(H,I)** Representative echocardiographic images from pulse-wave Doppler imaging **(H)** and tissue Doppler imaging **(I)** at the apical four-chamber view before and 12 h after LPS treatment. **(J)** Ratio of pulse-wave Doppler E wave to A wave amplitude. **(K)** Ratio of tissue Doppler E' wave to A' wave amplitude. *n* = 6 per group. Data represent means ± S.E.M. ^&^*p* < 0.05 vs. baseline; Statistical analyses were performed using two-way ANOVA followed by Bonferroni's *post-hoc* test. BAZ, Bazedoxifene.

Echocardiography was carried out to detect the alterations of cardiac function in mice after LPS treatment (10 mg/kg) for 12 h (Supplementary Tables S1, S2). Representative echocardiographic images from the parasternal short axis view were shown in Figure 1A. We found that LPS remarkably decreased LVEF (Figure 1B) and LVFS (Figure 1C), and increased LVIDs (Figure 1D) and LVESV (Figure 1F). LVIDd (Figure 1E) and LVEDV (Figure 1G) were increased in LPS group compared with control group but with no significance. Interestingly, Bazedoxifene improved the impairment in systolic function induced by LPS, including LVEF, LVFS, LVIDs, LVIDd, LVESV, and LVEDV. The cardiac diastolic function was also greatly impaired in LPS-induced sepsis. E/A ratio was remarkably reduced in LPS group (Figures 1H,J). Compared with control group, E'/A' ratio was decreased in LPS group but no statistical differences were found (Figures 1I,K). Noticeably, Bazedoxifene also prevented the diastolic dysfunction in LPS-treated mice, restoring E/A ratio and E'/A' ratio.

### Bazedoxifene Ameliorated Inflammation in LPS-Induced Sepsis

**Figure 2:**
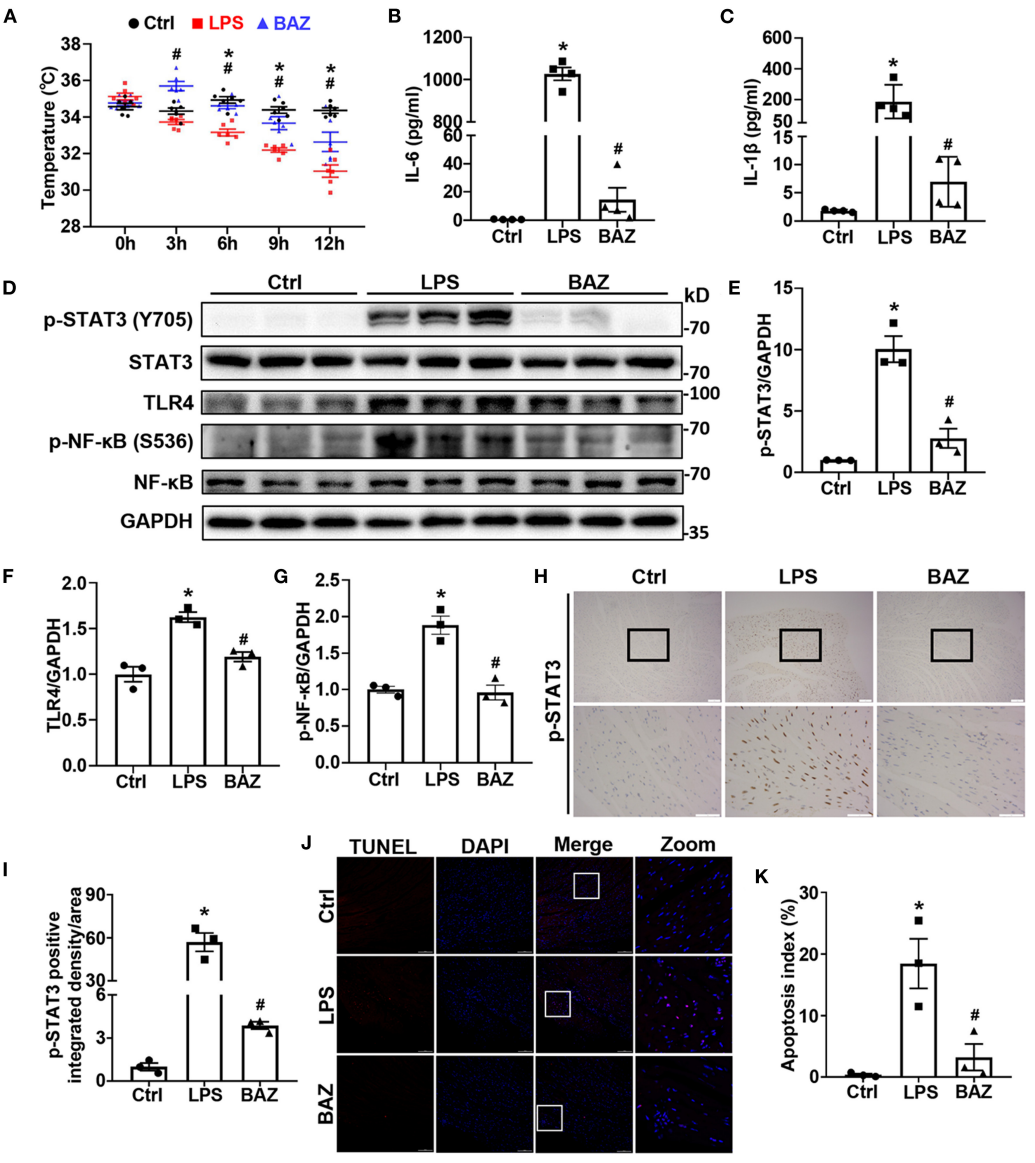
Bazedoxifene attenuated cardiac inflammatory and apoptosis levels in LPS-challenged mice. **(A)** Body temperature of mice at 0, 3, 6, 9, 12 h after LPS treatment. **(B,C)** ELISA assays were performed to measure IL-6 level **(B)** and IL-1β level **(C)** in mice serum. **(D–G)** Inflammatory signaling protein levels of p-STAT3 (Tyr705) **(E)**, STAT3, TLR4 **(F)**, p-NFκB (Ser536) **(G)**, and NFκB in mice heart tissues were determined by western blot analysis. **(H)** Representative immunohistochemical images of p-STAT3 (brown spots) in mice heart tissues. Upper scale bar: 100 μm; Lower scale bar: 50 μm. **(I)** Quantification of p-STAT3 positive area. **(J)** Representative images of TUNEL staining in mice heart tissues. Scale bar: 100 μm. **(K)** Quantification of apoptosis Index. *n* = 6 **(A)**, 4 **(B,C)**, and 3 **(D–K)** per group. Data represent means ± S.E.M. ^*^p < 0.05 vs. control; ^#^*p* < 0.05 vs. LPS treatment. Statistical analyses were performed using one-way ANOVA followed by Bonferroni's *post-hoc* test. BAZ, Bazedoxifene.

Compared with the control group, the body temperature was significantly decreased at 3, 6, 9, 12 h after LPS treatment, and treatment with Bazedoxifene ameliorated the alterations in body temperature (Figure 2A). Previous studies have shown that LPS increased both serum IL-6 and IL-1β levels, which were important indexes of LPS-induced damage (6, 16). Consistent with the literatures, LPS increased serum IL-6 and IL-1β levels (Figures 2B,C). Interestingly, Bazedoxifene remarkably decreased them. To examine the activation of STAT3 in LPS mouse heart, we checked the protein levels of p-STAT3 (Tyr705) and total STAT3 (Figure 2D). Our results revealed that LPS increased STAT3 phosphorylation with no significant alteration of total STAT3 (Figure 2E). TLR4 is a well-established membrane receptor for LPS (17), therefore we sought to investigate the effect of LPS to TLR4 and the downstream signaling of TLR4. As expected, LPS enhanced the levels of TLR4 and phosphorylation of NFκB (Ser536) without significant change of total NFκB (Figures 2D–G). Of note, Bazedoxifene attenuated enhanced STAT3 phosphorylation and TLR4/NFκB signaling to LPS stimulation. Immunohistochemical staining was also performed to verify the effects of LPS and Bazedoxifene on STAT3 in heart tissue (Figures 2H,I). As an additional index of damage, we used TUNEL staining to assess apoptosis of cardiomyocytes in each group. As shown in Figures 2J,K, few TUNEL positive cells in the control group whereas the percentage of TUNEL positive cells was dramatically increased in LPS group. Of note, the proportion of TUNEL positive cells was significantly reduced in BAZ group.

### Bazedoxifene Inhibited IL-6/STAT3 Signaling in Response to LPS in H9c2 Cells

**Figure 3:**
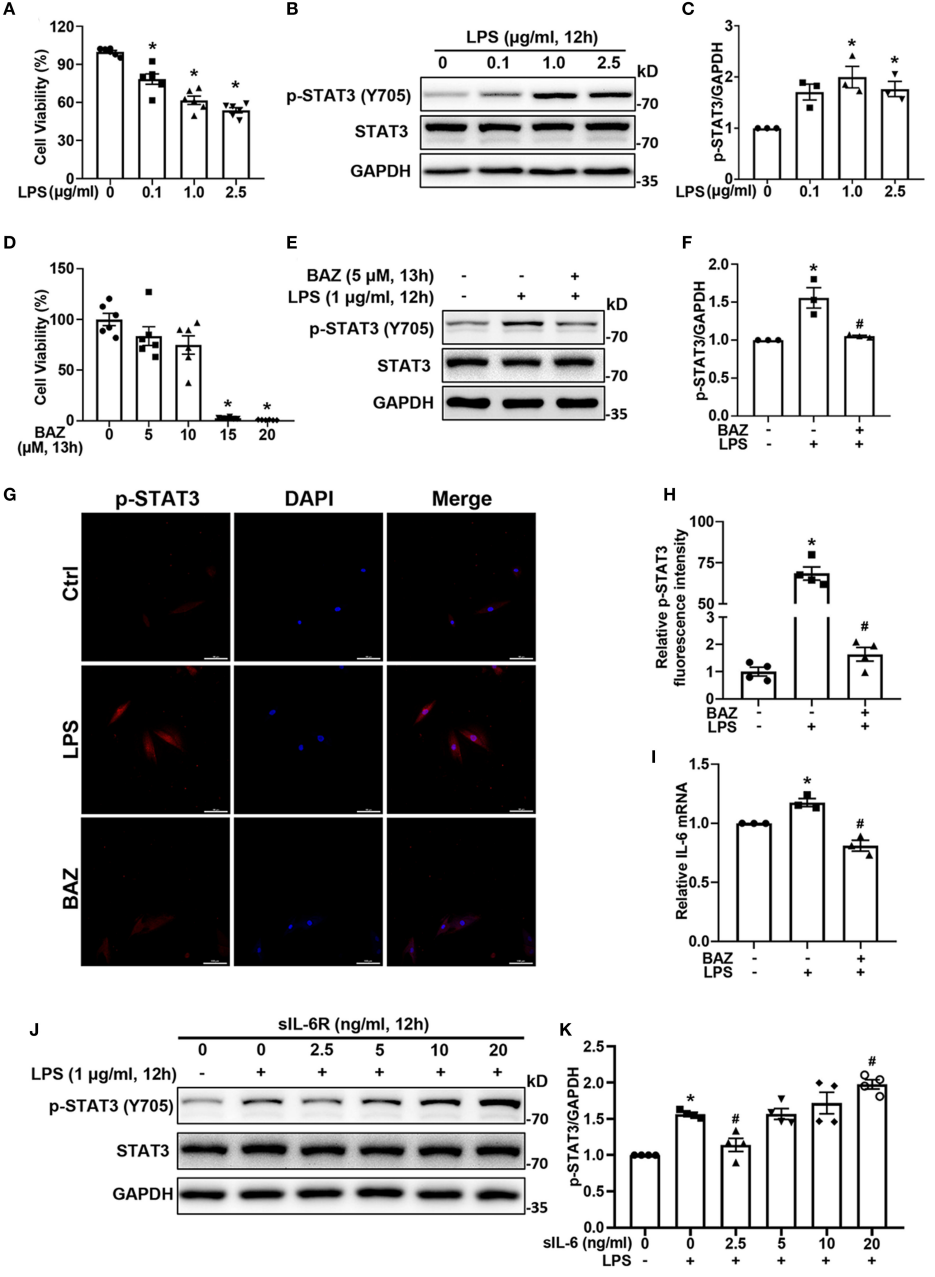
Bazedoxifene inhibited IL-6/STAT3 signaling induced by LPS in H9c2 cells. **(A)** Cell viability of H9c2 cells after treatment of various concentrations LPS for 12 h. **(B)** The corresponding STAT3 activation was determined by western blot analysis. **(C)** Quantitative analysis of western blot. **(D)** Cell viability of H9c2 cells after treatment of various concentrations Bazedoxifene for 13 h. **(E,F)**: For LPS stimuli, H9c2 cells were treated with LPS (1 μg/ml) for 12 h. For Bazedoxifene treatment, H9c2 cells were pretreated with 5 μM Bazedoxifene for 1 h and then stimulated by LPS (1 μg/ml) for another 12 h. Western blot was used to determine the levels of p-STAT3 and STAT3. **(G,H)** Representative immunofluorescence images and quantification for p-STAT3. Scale bar: 100 μm. **(I)** Quantification of IL-6 mRNA level. **(J,K)** The impact of various concentrations of sIL-6R on the activation of STAT3 induced by LPS. H9c2 cells were co-treated with LPS and sIL-6R (0, 2.5, 5, 10, 20 ng/ml) for 12 h. Representative western blot images and quantification of p-STAT3 and STAT3 were as indicated in the figure. *n* = 6 **(A,D)**, 3 **(B,C,E,F, I)**, and 4 **(G,H,J,K)** per group. Data represent means ± S.E.M. ^*^p < 0.05 vs. control; ^#^*p* < 0.05 vs. LPS treatment. Statistical analyses were performed using one-way ANOVA followed by Bonferroni's *post-hoc* test. BAZ, Bazedoxifene.

To explore the effects of LPS on cell viability, we treated H9c2 cells with various concentrations of LPS. LPS significantly decreased cell viability in a concentration-dependent manner (Figure 3A). In addition, the phosphorylation of STAT3 was induced by LPS (Figures 3B,C). Among them, 1.0 μg/ml LPS showed the strongest signal increase. To select the appropriate concentration of Bazedoxifene for further experiments, we also assessed the cytotoxicity of Bazedoxifene (Figure 3D). Therefore, 5 μM was chosen as the final concentration for further experiments. Bazedoxifene inhibited the induction of STAT3 phosphorylation by LPS, with no significant inhibition of total STAT3 (Figures 3E,F). Immunofluorescence analysis was also performed to verify the effect of LPS and Bazedoxifene on STAT3. Consistent with the western blot result, STAT3 phosphorylation caused by LPS was suppressed by Bazedoxifene (Figures 3G,H). In parallel, we found that LPS upregulated the level of IL-6 mRNA, which was inhibited by Bazedoxifene (Figure 3I). IL-6 can bind to sIL-6R with comparable affinity to that of membrane-bound IL-6R (mIL-6R). Low concentration sIL-6R binds with IL-6 to exert an antagonistic effect, whereas high concentration IL-6/sIL-6R complex could activates membrane gp130, causing subsequent phosphorylation of JAK/STAT3 (14, 18). We showed that p-STAT3 (Y705) first dropped and then rose in response to LPS with increasing sIL-6R concentration (Figures 3J,K), whereas the levels of p-STAT3 (S727) were not significantly altered (Supplementary Figure S1).

### STAT3 Is a Potential Transcription Factor for FUNDC1 in Cardiomyocytes

**Figure 4:**
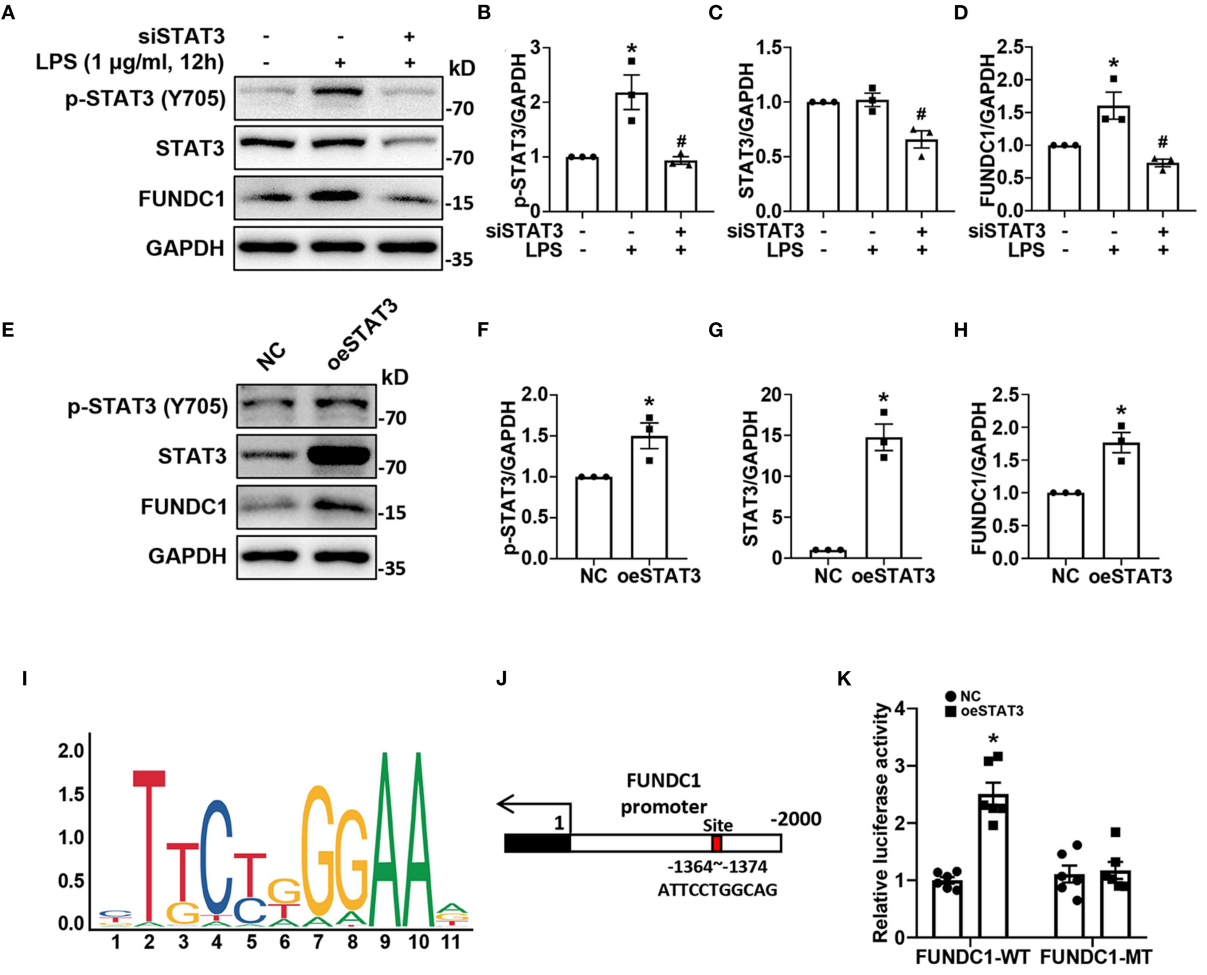
STAT3 promoted FUNDC1 expression through binding to the FUNDC1 promoter in cardiomyocytes. **(A–D)** H9c2 cells were transfected with control siRNA or STAT3 siRNA (siSTAT3), and then treated with LPS (1 μg/ml) for 12 h. P-STAT3 **(B)**, total STAT3 **(C)** and FUNDC1 **(D)** levels were examined by western blot. **(E–H)** AC16 cells were transfected with control plasmid or STAT3 overexpression plasmid (oeSTAT3), and then treated with LPS (1 μg/ml) for 12 h. P-STAT3 **(F)**, total STAT3 **(G)**, and FUNDC1 **(H)** levels were determined by western blot. **(I,J)** STAT3 motif **(I)** and STAT3-binding site **(J)** on FUNDC1 promoter. **(K)** AC16 cells were transfected with STAT3 overexpressed plasmid, wild-type (FUNDC1 WT) or mutant (FUNDC1 MT) FUNDC1 promoter plasmid for 48 h. Luciferase activity was measured using the dual-luciferase assay to detect the combined situation of STAT3 on FUNDC1 promoter in AC16 cells. *n* = 3 **(A–H)**, and 6 **(K)** per group. Data represent means ± S.E.M. ^*^p < 0.05 vs. control; ^#^*p* < 0.05 vs. LPS treatment. Statistical analyses were performed using one-way ANOVA followed by Bonferroni's *post-hoc* test **(B–D)**, unpaired Student's *t*-test **(F–H)**, and two-way ANOVA followed by Bonferroni's *post-hoc* test **(K)**. BAZ, Bazedoxifene.

We found that LPS treatment increased the protein levels of p-STAT3 and FUNDC1 in H9c2 cells, but had no effect on expression of total STAT3 (Figure 4A). In order to determine the effect of STAT3 on FUNDC1, we applied STAT3 siRNA (siSTAT3) to knock down STAT3 then checked the levels of FUNDC1 after LPS treatment. First, our results showed that siSTAT3 remarkably inhibited the levels of p-STAT3 and STAT3 (Figures 4B,C). Of note, the level of FUNDC1 induced by LPS was significantly inhibited by siSTAT3 (Figure 4D). Next, we used STAT3 overexpression plasmid (oeSTAT3) to overexpress STAT3 in AC16 cells and checked the protein levels of FUNDC1. Our results showed that STAT3 overexpression increased the levels of p-STAT3 and STAT3, with a concomitant increase in FUNDC1 expression (Figures 4E–H). It is well-known that STAT3 is a transcription factor; therefore, we speculated that STAT3 served as a transcription factor for FUNDC1. To test this hypothesis, we obtained the DNA motif of STAT3 and its binding site on FUNDC1 promoter from JASPAR website (http://jaspar.genereg.net/) (Figures 4I,J). Luciferase reporter assay results showed that STAT3 overexpression upregulated the activity of wild-type FUNDC1 promoter (FUNDC1 WT), whereas that of FUNDC1 promoter with mutant binding site (FUNDC1 MT) displayed no significant changes (Figure 4K).

### IL-6/STAT3 Inhibitor Suppressed MAMs Formation, Reduced Intracellular Ca^2+^ Levels and Expression of Calcium Signaling-Related Proteins in Response to LPS

**Figure 5:**
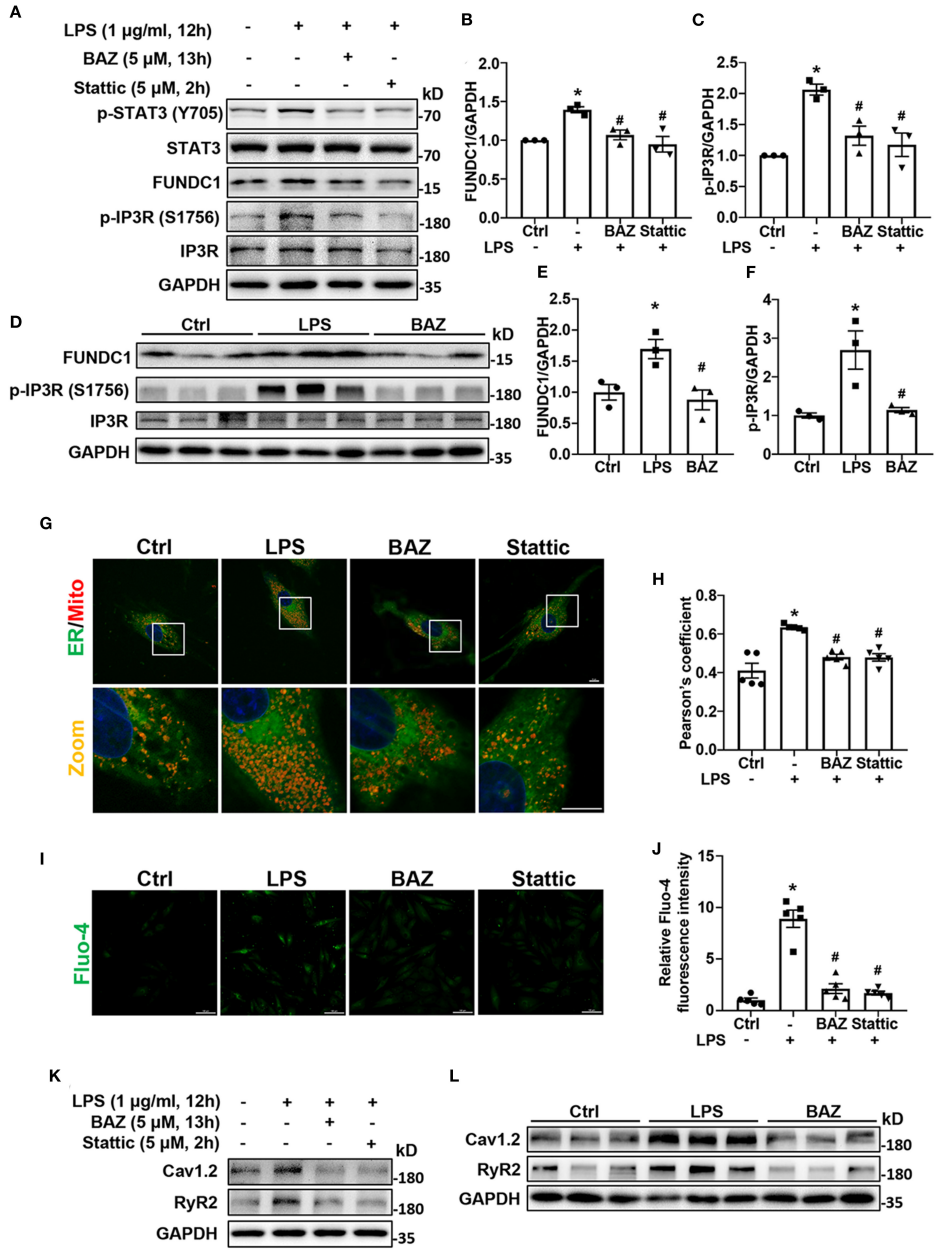
IL-6/STAT3 inhibitor attenuated LPS-induced mitochondria-associated endoplasmic reticulum (ER) membranes (MAMs) formation, intracellular Ca^2+^ levels, and expression of calcium signaling-related proteins in H9c2 cells. For LPS stimuli, H9c2 cells were treated with LPS (1 μg/ml) for 12 h. For BAZ group, H9c2 cells were pretreated with 5 μM Bazedoxifene for 1 h and stimulated by LPS (1 μg/ml) for another 12 h. H9c2 cells were pretreated with 5 μM Stattic for 2 h and then the culture medium was replaced with fresh medium containing LPS for another 12 h as the Stattic group. **(A–F)** The levels of MAM-associated proteins FUNDC1 **(B,E)** and p-IP3R (Ser 1756) **(C,F)** in H9c2 cells and mice heart tissues were determined by western blot analysis. **(G)** Contact between the ER and mitochondria (Mito) was evaluated by confocal microscopy. Representative images were shown. Scale bar: 10 μm. **(H)** Pearson's coefficient was quantified for detecting the association between ER and mitochondria. **(I)** Fluo-4 staining was performed to show intracellular Ca^2+^ levels. Scale bar: 50 μm. **(J)** Quantification of Fluo-4 fluorescence intensity. **(K,L)** The levels of calcium signaling-related proteins Cav1.2 and RyR2 in H9c2 cells **(K)** and mice heart tissues **(L)** were determined by western blot analysis. *n* = 3 **(A–F,K,L)**, and 5 **(G–J)** per group. Data represent means ± S.E.M. ^*^p < 0.05 vs. control; ^#^*p* < 0.05 vs. LPS treatment. Statistical analyses were performed using one-way ANOVA followed by Bonferroni's *post-hoc* test. BAZ, Bazedoxifene.

In order to determine the effect of LPS on MAMs formation, we first checked the levels of MAM-associated proteins, including FUNDC1, phosphorylation of IP3R, and total IP3R by western blot in H9c2 cells (Figure 5A). The levels of FUNDC1 and phosphorylation of IP3R (Ser 1756) were significantly higher in LPS-treated H9c2 cells, with no change in total protein level of IP3R (Figures 5B,C). Next, we used Bazedoxifene to investigated the effects of IL-6/STAT3 inhibition on LPS-induced MAM-related proteins alterations. Our results revealed that Bazedoxifene dramatically inhibited the levels of FUNDC1 and IP3R phosphorylation induced by LPS. Then we utilized a specific STAT3 inhibitor, Stattic to prove the functions of STAT3 in LPS treatment. Similarly, Stattic inhibited the levels of MAM-associated proteins FUNDC1 and p-IP3R in LPS-induced H9c2 cells (Figures 5B,C). To test whether these effects can also be observed *in vivo*, we determined the proteins levels in mice heart homogenates (Figure 5D). As expect, increased levels of both FUNDC1 and p-IP3R were detected in LPS-treated mice and Bazedoxifene inversed these effects (Figures 5E,F). Moreover, we used confocal imaging and Pearson's coefficient to evaluate the MAMs formation (Figures 5G,H). We found that contact sites were increased in LPS-treated H9c2 cells and IL-6/STAT3 inhibitor Bazedoxifene and Stattic reversed the effect.

Increased MAMs formation can contribute to increased intracellular Ca^2+^ levels (12). Next, we used fluorescent probe Fluo-4 to detect intracellular Ca^2+^ levels. As expected, the LPS-induced increases in intracellular Ca^2+^ levels were observed in H9c2 cells, which was inhibited by IL-6/STAT3 inhibitor (Figures 5I,J).

Because of the increased intracellular Ca^2+^ levels after LPS treatment, we further sought to determine whether the levels of calcium signaling-related proteins were altered, including Cav1.2 and RyR2. Our results revealed that LPS induced the increases of Cav1.2 and RyR2 levels in H9c2 cells, which were suppressed by Bazedoxifene and Stattic (Figure 5K; Supplementary Figures S2A,B). Next, we also found the protein levels of Cav1.2 and RyR2 were upregulated in LPS-treated mice heart and Bazedoxifene downregulated Cav1.2 and RyR2 levels induced by LPS (Figure 5L; Supplementary Figures S2C,D).

### IL-6/STAT3 Inhibitor Alleviated Mitochondrial Dysfunction and ROS Production in Response to LPS

**Figure 6:**
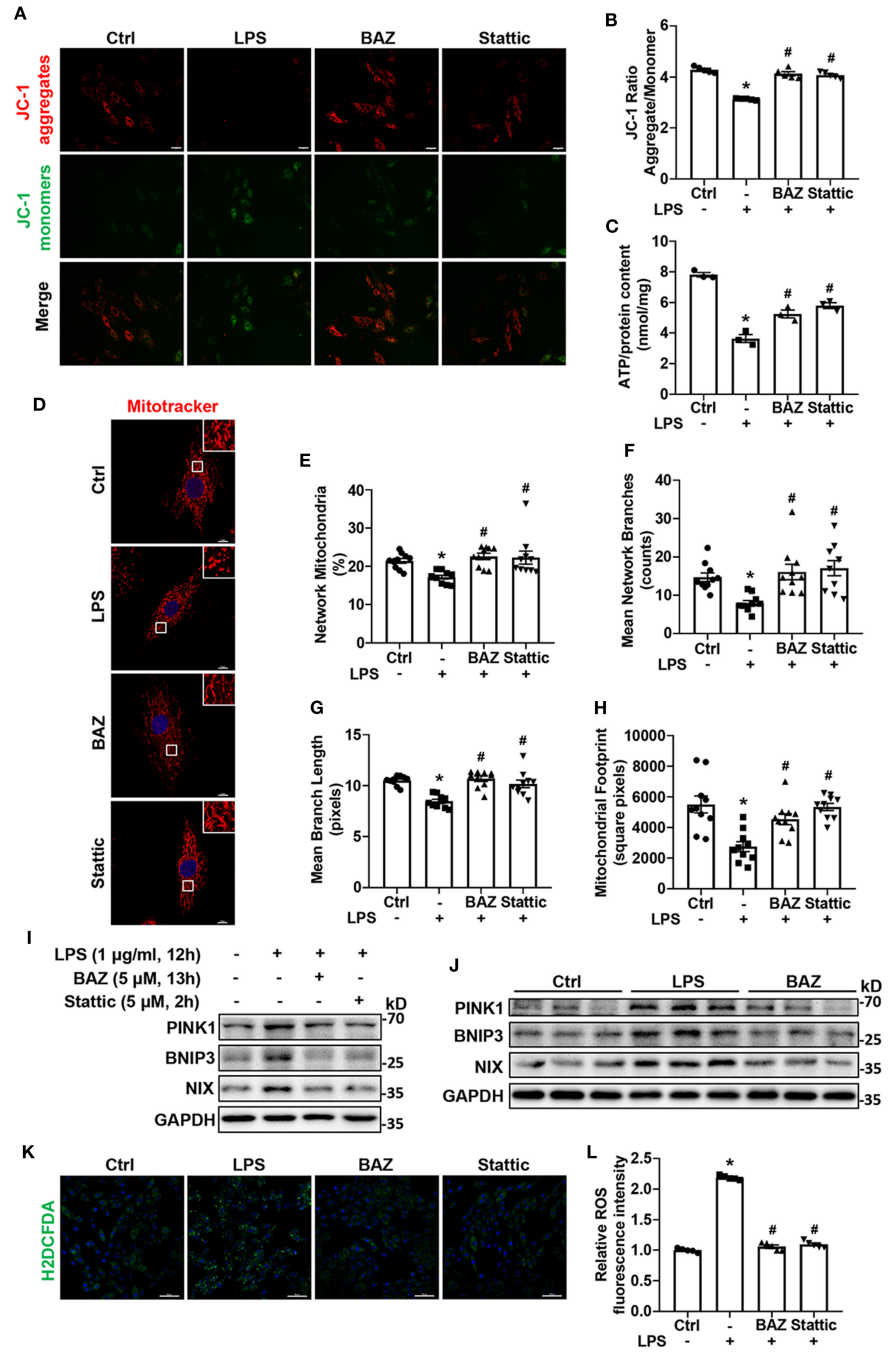
IL-6/STAT3 inhibitor alleviated LPS-challenged mitochondrial dysfunction and ROS production in cardiomyocytes. H9c2 cells were treated as described in Figure 5. **(A)** JC-1 staining was used to detect the mitochondrial membrane potential. Representative fluorescence images of JC-1 aggregate (red) and JC-1 monomer (green) are shown in the figure. Scale bar: 50 μm. **(B)** Quantification of mitochondrial membrane potential (JC-1 aggregate/ monomer ratio). **(C)** ATP content was measured in each group. **(D)** We used Mito-Tracker Red dye to determine the mitochondrial morphology. Representative images were presented in the figure. Scale bar: 10 μm. **(E–H)** The ImageJ macro tool MiNA was utilized to analyze mitochondrial network morphology, including percentage of network mitochondria **(E)**, mean number of mitochondrial branches **(F)**, mean branch length **(G)**, and mitochondrial footprint **(H)**. **(I,J)** Representative western blot images of mitophagy proteins PINK1, BNIP3, and NIX in H9c2 cells **(I)** and mice heart tissues **(J)**. **(K)** H2DCFDA staining was used to detect ROS production. Scale bar: 100 μm. **(L)** Quantification of H2DCFDA fluorescence intensity for ROS production. *n* = 5 **(A,B,K,L)**, 3 **(C,I,J)**, and 10 **(D–H)** per group. Data represent means ± S.E.M. ^*^p < 0.05 vs. control; ^#^*p* < 0.05 vs. LPS treatment. Statistical analyses were performed using one-way ANOVA followed by Bonferroni's *post-hoc* test. BAZ, Bazedoxifene.

MAMs formation can impact mitochondrial function and ROS production. To explore the effect of LPS on mitochondrial function, we used JC-1 staining to determine Δψm. LPS treatment reduced JC-1 aggregate/monomer ratio in H9c2 cells, which indicated Δψm loss. IL-6/STAT3 inhibitor Bazedoxifene and Stattic restored LPS-induced Δψm alteration (Figures 6A,B). ATP content is also used to assess mitochondrial function. We found that LPS enormously decreased the level of ATP, which was ameliorated by Bazedoxifene and Stattic (Figure 6C). To further evaluate mitochondrial function, we performed Mito-tracker Red staining to observe mitochondrial network morphology. The results verified that LPS caused mitochondrial fragmentation (Figure 6D), which was assessed by percentage of network mitochondria (Figure 6E), mean number of mitochondrial branches (Figure 6F), mean branch length (Figure 6G), and mitochondrial footprint (Figure 6H). IL-6/STAT3 inhibitor Bazedoxifene and Stattic reversed LPS-induced mitochondrial fragmentation. Mitochondrial fragmentation and dysfunction can induce mitophagy. Therefore, we checked the levels of mitophagy proteins by western blot. Compared with the control group, we found LPS increased the expression of mitophagy proteins including BNIP3, NIX, and PINK1 and these effects could be inhibited by Bazedoxifene and Stattic *in vitro* (Figure 6I; Supplementary Figures S3A–C). Consistent with the results *in vitro*, LPS caused the increased mitophagy proteins in heart tissue homogenates of LPS-treated mice, which was restored by Bazedoxifene treatment (Figure 6J; Supplementary Figures S3D–F). It is well-known that mitochondrial dysfunction contributed to oxidative stress (19, 20). Next, we checked the level of intracellular ROS using the fluorescent dye H2DCFDA in H9c2 cells. As expected, ROS generation was remarkably enhanced upon exposure to LPS (Figures 6K,L). Of note, the increased ROS levels were largely rescued by Bazedoxifene and Stattic.

### Disrupting MAMs by FUNDC1-Knockdown Prevented LPS-Induced Increases of Intracellular Ca^2+^ Levels and Expression of Calcium Signaling-Related Proteins, Mitochondrial Dysfunction, and ROS Production in H9c2 Cells

**Figure 7:**
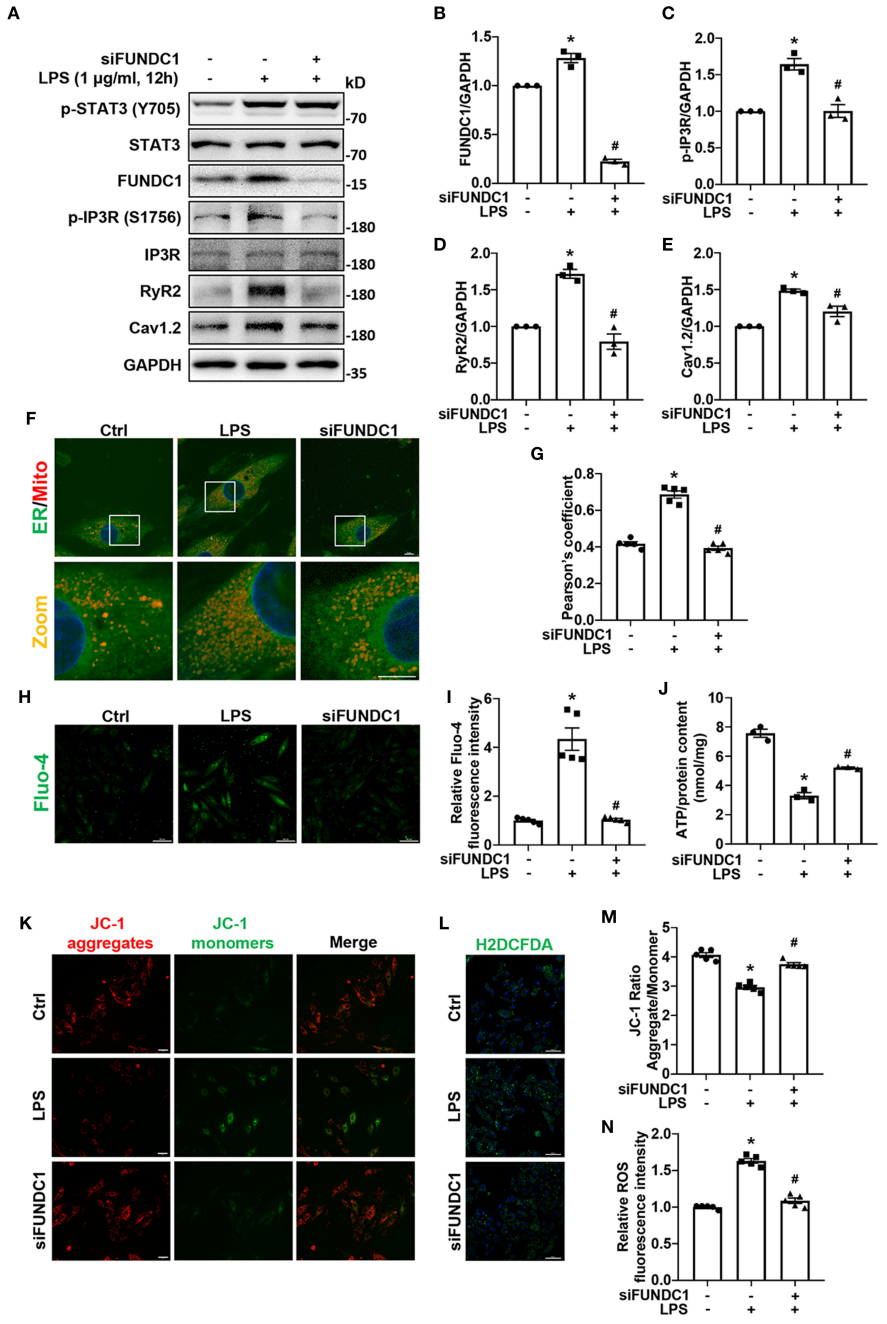
FUNDC1-knockdown prevented LPS-induced calcium signaling-related proteins expression, MAMs formation, increases of intracellular Ca^2+^ levels, mitochondrial dysfunction, and ROS production. H9c2 cells were transfected with control siRNA or FUNDC1 siRNA (siFUNDC1), and then treated with LPS (1 μg/ml for 12 h. **(A–E)** FUNDC1 **(B)**, p-IP3R (Ser 1756) **(C)**, IP3R, RyR2 **(D)**, and Cav1.2 **(E)** levels were examined by western blot. **(F,G)** Representative confocal images **(F)** and quantification **(G)** of ER-mitochondria contacts. Scale bar: 10 μm. **(H,I)** Representative images **(H)** and fluorescence intensity quantification **(I)** of Fluo-4 staining. Scale bar: 100 μm. **(J)** ATP content in each group. **(K)** Representative images of JC-1 staining. Scale bar: 50 μm. **(L)** Representative images of H2DCFDA staining. Scale bar: 100 μm. **(M)** Quantification of the JC-1 fluorescence ratio (JC-1 aggregate/JC-1 monomer). **(N)** Quantification of ROS production as measured by H2DCFDA fluorescence intensity. *n* = 3 **(A–E,J)** and 5 **(F–I,K–N)** per group. Data represent means ± S.E.M. ^*^p < 0.05 vs. control; ^#^*p* < 0.05 vs. LPS treatment. Statistical analyses were performed using one-way ANOVA followed by Bonferroni's *post-hoc* test. BAZ, Bazedoxifene.

To identify the role of FUNDC1 in LPS-induced myocardial injury, we utilized FUNDC1 siRNA (siFUNDC1) to knock down FUNDC1 in H9c2 cells and observed the effect of siFUNDC1 on MAMs formation, expression of calcium signaling-related proteins, intracellular Ca^2+^ levels, mitochondrial function, and ROS production after LPS treatment. We found that FUNDC1 knockdown did not impact the activation of STAT3 induced by LPS (Figure 7A; Supplementary Figure S4). We first checked the expression of MAM-associated proteins and calcium signaling-related proteins by western blot (Figure 7A). We found that siFUNDC1 significantly downregulated LPS-induced FUNDC1 expression (Figure 7B). Consistent with IL-6/STAT3 inhibitor, FUNDC1 deletion inhibited the phosphorylation of IP3R (Ser 1756) induced by LPS, with no significant alteration of total IP3R (Figure 7C). Interestingly, LPS-induced upregulation of RyR2 and Cav1.2 levels were also suppressed by FUNDC1 knockdown (Figures 7D,E). As expected, siFUNDC1 alleviated LPS-induced MAMs formation (Figures 7F,G), intracellular calcium overload (Figures 7H,I), reduction of ATP levels (Figure 7J), loss of Δψm (Figures 7K,M), and ROS production (Figures 7L,N). Taken together, FUNDC1 plays an important role in LPS-induced myocardial dysfunction.

## Discussion

Although sepsis can affect many important organs, SIMD is the major cause of mortality and morbidity during septic shock (4). At present, there are no suggested guidelines or therapeutic strategies for SIMD. Because of its multifactorial and unclear pathogenesis, there are no specific medications (21). In the present study, we have demonstrated the crucial role of IL-6/STAT3 signaling in LPS-induced myocardial dysfunction. IL-6/STAT3 inhibitor Bazedoxifene prevented myocardial inflammation, cardiac apoptosis, and cardiac systolic and diastolic dysfunction induced by LPS. Here, we first revealed that STAT3 was the potential transcription factor of FUNDC1, and STAT3 might lead to LPS-induced myocardial injury through regulating the FUNDC1-dependent MAMs formation.

Cardiac dysfunction is one of the most common and severe complications for patients with sepsis, which is characterized by an overall (systolic and diastolic) dysfunction of both left and right heart (22). Several studies find that both LPS treatment and cecal ligation and puncture significantly inhibit myocardial function, commonly presenting as decreased ejection fraction, ventricular dilatation, and diastolic dysfunction (23, 24). Consistent with this finding, our results showed that LPS challenge remarkably decreased LVEF and LVFS, increased left ventricular diameter and volume, and inhibited E/A ratio and E'/A' ratio. Previous research (25) shows that survival rate of sepsis mice at 7 days is about 20%, whereas improvement in cardiac function at 12 h brings up the survival to 75%. Excitingly, we found that Bazedoxifene enormously ameliorated cardiac dysfunction induced by LPS at 12 h, which might suggest a good prognosis.

One of characteristics for early sepsis is the excessive inflammation syndrome called “cytokine storm” (26). IL-6 is one of the most commonly used biomarkers of sepsis, and the level of IL-6 is positively correlated with sepsis-associated organ dysfunction and death (8, 27). Similarly, the role of IL-6 has been demonstrated in septic animal models induced by cecal ligation and puncture or LPS (28, 29). The latest study also shows that stress-induced IL-6 is required to enhance end-organ injury and mortality caused by LPS (30). STAT3 is the main downstream effector of IL-6 signaling. Zhang et al. (31) and Zhen et al. (11) show that melatonin and traditional Chinese medicine Oxymatrine relieve sepsis-induced myocardial injury and simultaneously inhibit STAT3 signaling. However, it is still not fully understood that whether STAT3 plays a direct role and the specific mechanisms by which STAT3 contributes to SIMD. Here, we found that IL-6/STAT3 signaling was remarkably activated in LPS-treated mice heart tissues and cultured cells. IL-6/STAT3 inhibitor alleviated LPS-induced cardiac dysfunction, intracellular calcium overload, mitochondrial dysfunction, and ROS production.

Gao et al. (32) reveals that IL-6 level in the supernatant of H9c2 cells is significantly increased in response to LPS. Similarly, we treated H9c2 cells with LPS and found that LPS induced the expression of IL-6 mRNA, which should be attributed to the activation of NF-κB (9). Notably, Bazedoxifene inhibited the expression of IL-6 mRNA after LPS treatment. We therefore speculated that SIMD might be attributed to the IL-6 from both circulating blood and local myocardium. STAT3 is a transcription factor activated by multiple stimuli including IL-6, IL-10, IL-17, VEGF, TGF-β, LIF, and so on (33). Therefore, we introduced the sIL-6R to demonstrate that LPS-induced STAT3 activation is associated with IL-6. IL-6 signaling can active STAT3 through either mIL-6R (classical pathway) or sIL-6R (trans-signaling pathway) (9). We found the level of p-STAT3 changed depending on sIL-6R concentration in the presence of LPS, which indicated the LPS-induced STAT3 activation might be partially mediated by IL-6.

Mitochondria interact tightly with other organelles in cell, especially the ER. MAMs are the physically and functionally contact sites between mitochondria and ER. MAMs play an important role in several cardiac diseases, such as diabetic cardiomyopathy, heart failure, and obesity cardiomyopathy (34–36). FUNDC1 is the newly discovered MAMs protein and related to calcium transmission, mitochondrial function and ROS production. Our results showed that the expression of FUNDC1 was dramatically increased in LPS-treated mice heart and H9c2 cells, which was inhibited by IL-6/STAT3 inhibitor. Moreover, STAT3 knockdown or overexpression positively regulated FUNDC1 expression. Using the JASPAR database, we found that STAT3 binding site was present in the FUNDC1 promoter region. Dual luciferase reporter assay was performed to confirm this finding. To our knowledge, we presented for the first time that STAT3 is the potential transcription factor for FUNDC1. FUNDC1 is also a mitophagy protein with LC3-interacting region. Phosphorylation of FUNDC1 at Tyr18 and Ser13 can impact the interaction between FUNDC1 and LC3II, and then regulates mitophagy (37). We have found that alteration in the expression level of FUNDC1 is important in MAMs formation, however, what role of FUNDC1 phosphorylation is unknown. There are no studies about the role of FUNDC1 phosphorylation in MAMs formation, so it deserves further investigation. Jiang et al. (38) reveal that FUNDC1-induced mitophagy serves a key role in LPS-treated H9c2 cells. Interestingly, the authors find the level of FUNDC1 is decreased after LPS treatment. With regard to this, it is possibly related to different phases of cell damage. The authors check the effects of LPS at 24 h, whereas we focused on earlier stage (12 h). Further studies should pay attention to all phases of sepsis and develop targeted interventions at different stages.

IP3R is an ER resident ligand-gated Ca^2+^ channel, which binds to IP3, triggering the release of Ca^2+^ from ER (39). Recent researches indicate that FUNDC1 forms a protein bridge with IP3R to localize in MAMs (12, 34, 35). Our data showed that LPS did not change the protein level of IP3R, but dramatically increased the phosphorylation of IP3R at Ser1756, which was ameliorated by IL-6/STAT3 inhibitor and FUNDC1 knockdown. Ser1756 phosphorylation of IP3R enhances the sensitivity to IP3 then increases the release of Ca^2+^ from ER (40). In parallel, we utilized fluorescence co-localization to determine the formation of MAMs and found that IL-6/STAT3 inhibitor and FUNDC1 knockdown suppressed LPS-induced MAMs formation. Intracellular Ca^2+^ increase is a major characteristic of increased MAMs formation. IL-6/STAT3 inhibitor and knockdown of FUNDC1 ameliorated LPS-induced intracellular calcium overload. There are many other proteins participating in the regulation of intracellular Ca^2+^ levels, such as Cav1.2 and RyR2. Cav1.2 is the primary pore-forming subunit of L-type calcium channels in cardiomyocytes and selectively allows the passage of calcium ions then affects the contraction of the cardiac muscle (41). RyR2 is one of Ca^2+^ release channels in ER that plays an essential role in the contraction of cardiac myocytes (42). Our data revealed the levels of Cav1.2 and RyR2 were increased after LPS treatment. In addition, IL-6/STAT3 inhibitor and FUNDC1-knockdown reversed the protein levels of RyR2 and Cav1.2 induced by LPS, which suggested FUNDC1-dependent MAMs formation might affect the intracellular Ca^2+^ levels by partially influencing Cav1.2 and RyR2 levels.

MAMs serve as an important role in SIMD. This is consistent with the literature by Sun et al. (43); however, their findings suggest that treatment of LPS reduced the level and activity of MAMs. In this regard, we suspect that it is related to different stages of disease development during sepsis. They check the MAMs formation at 18 h post-LPS treatment, the late infection stage. At this time, cardiomyocytes exhibit severe injuries and the structure of MAMs may be damaged. Together, FUNDC1-dependent MAMs is important in LPS-induced sepsis and IL-6/STAT3 inhibitor might improve cardiac dysfunction through regulating the FUNDC1-dependent MAMs formation. It was a significant breakthrough in understanding the role of IL-6/STAT3 axis in acute inflammatory disease.

Raised intracellular Ca^2+^ mediated by MAMs can contribute to mitochondrial dysfunction (44). From our data, mitochondrial membrane potential, ATP production, and mitochondrial network morphology were impaired after LPS stimuli, which were reversed by IL-6/STAT3 inhibitor and FUNDC1 knockdown. Mitophagy, a selective autophagy of mitochondria that responses to mitochondrial dysfunction promotes mitochondrial quality control (45). Our results revealed that LPS triggered the expression of PINK1, the classical mitophagy pathway protein. In addition to classical mitophagy pathway, the receptor of MOM can also mediate mitophagy. NIX and BNIP3 are well-studied MOM receptors with LC3-interacting region (46). We found that the levels of NIX and BNIP3 were significantly increased after LPS stimulation. Mitophagy is reported to play a favorable role in sepsis, because of the clearance of disabled mitochondria (45). However, we note that the majority of studies check the effects of sepsis beyond 24 h. Patoli et al. (47) show that inhibition of mitophagy improves the outcome of sepsis at early time points. It is also reported that excessive mitophagy can lead to cell apoptosis in several cardiac diseases, such as heart failure, myocardial anoxia/reoxygenation injury, and obesity-related metabolic heart disease (10, 48, 49). Of note, IL-6/STAT3 inhibitor alleviated the excessive expression of PINK1, NIX, and BNIP3. Hence, moderate mitophagy is necessary for mitochondrial health and cell function. Moreover, our results revealed that IL-6/STAT3 inhibitor and knockdown of FUNDC1 also significantly ameliorated ROS production induced by LPS. Thus, inhibition of IL-6/STAT3/FUNDC1 pathway could attenuate LPS-induced mitochondrial dysfunction and ROS production.

Inhibition of IL-6 signaling is considered as a promising therapeutic strategy. Siltuximab and tocilizumab, targeting IL-6 and the IL-6R respectively, have been approved for the treatment of Castleman disease (siltuximab) and arthritis (tocilizumab). In addition, there are a large number of clinical trials targeting IL-6/STAT3 pathway are currently in progress (50, 51). Bazedoxifene is the inhibitor of IL-6/gp130 protein-protein interactions and our present studies find that Bazedoxifene can influence IL-6/gp130 interface and then inhibits IL-6/STAT3 signaling pathway in tumor and cardiovascular disease (14, 15, 52). Bazedoxifene has been used in the clinics for osteoporotic bones in postmenopausal women, and our findings may provide new insights into the indications of Bazedoxifene. However, some limitations should be mentioned. In this study, Bazedoxifene was applied prior to LPS challenge. However, post-treatment is more applicable in the clinic and thus post-treatment of Bazedoxifene in sepsis is worth pursuing in future research. We used LPS to establish the sepsis model, but it cannot fully simulate human sepsis. In present study, we primarily investigated the function of FUNDC1-dependent MAMs. It is possible that other MAMs-related proteins may also play a role. Therefore, further studies were needed to illustrate the function of other MAMs-related proteins in LPS-treated myocardium.

## Conclusions

In summary, we have identified an important role of IL-6/STAT3 axis in SIMD, through regulating FUNDC1-dependent MAMs formation and interfering the function of mitochondria and ER. The finding is important to clarify the mechanism of SIMD, and IL-6/STAT3/FUNDC1 could be a novel promising target for treatment of SIMD.

## Data Availability Statement

The raw data supporting the conclusions of this article will be made available by the authors, without undue reservation.

## Ethics Statement

The animal study was reviewed and approved by the Animal Care and Use Committee of Tongji Hospital, Tongji Medical College, Huazhong University of Science and Technology.

## Author Contributions

LL, JL, and TJ conceived and designed the work. TJ performed most of the experiments, analyzed data, and wrote the manuscript. DP, WS, JG, SH, and LM helped with experiments and data analysis. CZ and SL provided essential reagents, equipment, and advice. LL and JL provided feedback on experiments, reviewed, and edited the manuscript. All authors contributed to the article and approved the submitted version.

## Funding

This work was supported by the Science and Technology Project Foundation of Wuhan (Grant Number 2019020701011439) and the National Natural Science Foundation of China (Grant Numbers 82070396 and 81974032).

## Conflict of Interest

The authors declare that the research was conducted in the absence of any commercial or financial relationships that could be construed as a potential conflict of interest.

## Publisher's Note

All claims expressed in this article are solely those of the authors and do not necessarily represent those of their affiliated organizations, or those of the publisher, the editors and the reviewers. Any product that may be evaluated in this article, or claim that may be made by its manufacturer, is not guaranteed or endorsed by the publisher.
