# Supplementary material for: IL-6/STAT3 Signaling Promotes Cardiac Dysfunction by Upregulating FUNDC1-Dependent Mitochondria-Associated Endoplasmic Reticulum Membranes Formation in Sepsis Mice
Source: Front Cardiovasc Med. 2022 Jan 18;8:790612. doi: 10.3389/fcvm.2021.790612 (PMC8804492; doi:10.3389/fcvm.2021.790612)
Supplement: Supplementary file 1 [file Data_Sheet_1.docx]

Supplementary Material

| **Supplementary Table1: Echocardiographic parameters at baseline:** | | | | |
| --- | --- | --- | --- | --- |
|  |  | **Ctrl (n=6)** | **LPS (n=6)** | **BAZ (n=6)** |
| **LVEF** | **%** | **76.80±5.45** | **75.02±3.17** | **76.10±3.50** |
| **LVFS** | **%** | **40.04±4.85** | **38.29±2.77** | **39.28±3.12** |
| **LVIDs** | **mm** | **2.32±0.26** | **2.45±0.14** | **2.45±0.15** |
| **LVIDd** | **mm** | **3.87±0.25** | **3.96±0.14** | **4.04±0.26** |
| **LVESV** | **mL** | **0.03±0.01** | **0.04±0.01** | **0.04±0.01** |
| **LVEDV** | **mL** | **0.15±0.03** | **0.16±0.02** | **0.17±0.03** |
| **E/A Ratio** |  | **1.98±0.27** | **1.84±0.43** | **1.98±0.48** |
| **E’/A’ Ratio** |  | **1.52±0.38** | **1.62±0.28** | **1.60±0.34** |
| **Data are presented as mean±SD.** | | | | |

| **Supplementary Table2: Echocardiographic parameters after treatment:** | | | | |
| --- | --- | --- | --- | --- |
|  |  | **Ctrl (n=6)** | **LPS (n=6)** | **BAZ (n=6)** |
| **LVEF** | **%** | **74.95±3.03** | **44.20±5.80** | **68.11±1.66** |
| **LVFS** | **%** | **38.23±2.57** | **18.53±2.91** | **32.37±1.15** |
| **LVIDs** | **mm** | **2.46±0.22** | **3.49±0.53** | **2.38±0.12** |
| **LVIDd** | **mm** | **3.98±0.30** | **4.28±0.61** | **3.52±0.20** |
| **LVESV** | **mL** | **0.04±0.01** | **0.11±0.05** | **0.04±0.01** |
| **LVEDV** | **mL** | **0.16±0.03** | **0.20±0.08** | **0.11±0.02** |
| **E/A Ratio** |  | **1.82±0.16** | **0.80±0.34** | **1.48±0.16** |
| **E’/A’ Ratio** |  | **1.57±0.43** | **0.98±0.34** | **1.54±0.31** |
| **Data are presented as mean±SD.** | | | | |


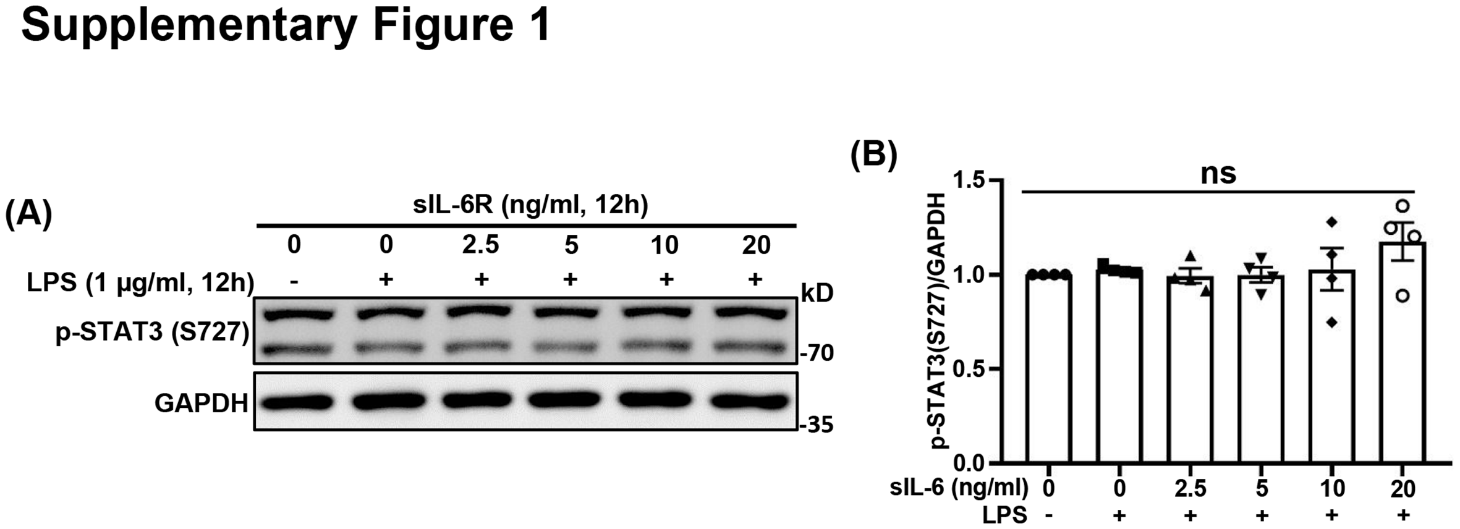


**Figure S1.** **A-B:** Representative western blot images (A) and quantification of p-STAT3 (S727) (B). n = 4 per group. Data represent means ± S.E.M. Statistical analyses were performed using one-way ANOVA followed by Bonferroni’s post-hoc test.


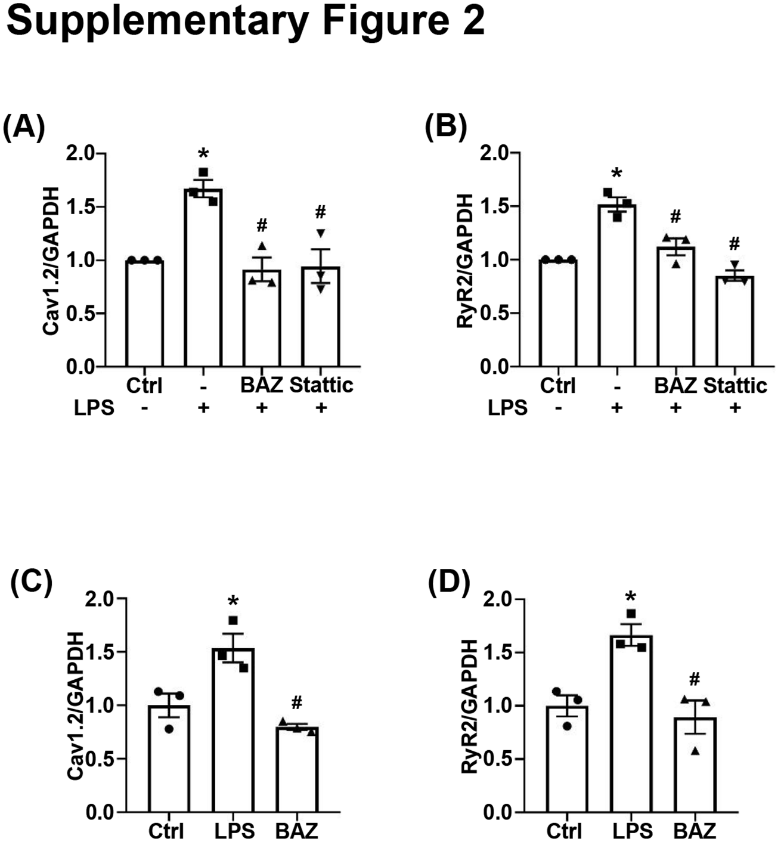


**Figure S2. A-B:** Quantification of Cav1.2 (A) and RyR2 (B) in Figure 5K. **C-D:** Quantification of Cav1.2 (C) and RyR2 (D) in Figure 5L. n = 3 per group. Data represent means ± S.E.M. **p* < 0.05 vs control; ^#^*p* < 0.05 vs LPS treatment. Statistical analyses were performed using one-way ANOVA followed by Bonferroni’s post-hoc test. BAZ, Bazedoxifene.


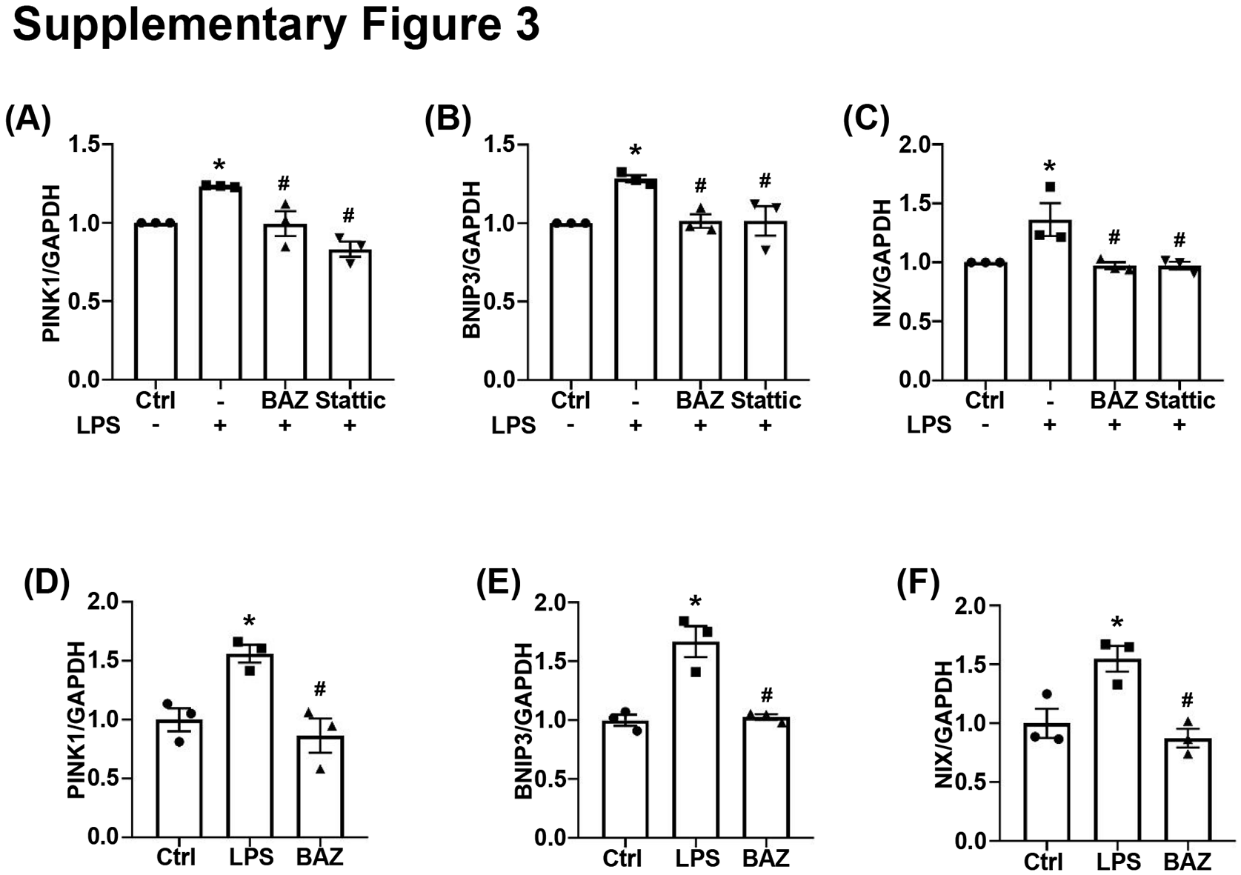


**Figure S3. A-C:** Quantification of PINK1 (A), BNIP3 (B), and NIX (C) in Figure 6I. **C-D:** Quantification of PINK1 (D), BNIP3 (E), and NIX (F) in Figure 6J. n = 3 per group. Data represent means ± S.E.M. **p* < 0.05 vs control; ^#^*p* < 0.05 vs LPS treatment. Statistical analyses were performed using one-way ANOVA followed by Bonferroni’s post-hoc test. BAZ, Bazedoxifene.


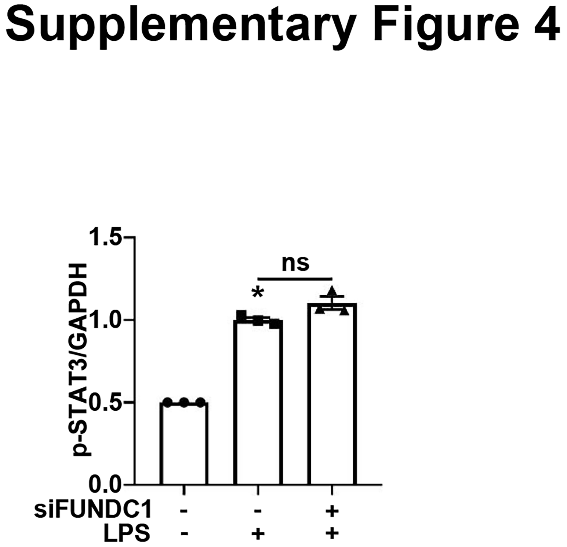


**Figure S4.** Quantification of p-STAT3 (Y705) in Figure 7A. n = 3 per group. Data represent means ± S.E.M. **p* < 0.05 vs control; ns = not significant. Statistical analyses were performed using one-way ANOVA followed by Bonferroni’s post-hoc test.
